# Evaluating the learning curve and operative time of interlaminar and transforaminal endoscopic lumbar discectomy

**DOI:** 10.1016/j.bas.2025.104225

**Published:** 2025-02-25

**Authors:** Youssef Jamaleddine, Ahmad Haj Hussein, Mohamad Omar Honeine, Elio Daccache, Sarah El Hajjar, Ramzi Moucharafieh, Nizar Natout, Mohammad Badra

**Affiliations:** a Gilbert and Rose-Marie Chagoury School of Medicine, Lebanese American University, Beirut, Lebanon; b Department of Orthopedic Surgery, Faculty of Medicine, Balamand University, Beirut, Lebanon; c Department of Orthopedics and Traumatology, Clemenceau Medical Center, Beirut, Lebanon

**Keywords:** Discectomy, Endoscopic, Operative, Time, Learning, Curve

## Abstract

**Introduction:**

Lumbar disc herniation is common in spine surgery, and endoscopic lumbar discectomy (ELD) offers a minimally invasive alternative with reduced complications. However, the learning curve of ELD, particularly between interlaminar and transforaminal techniques, remains a challenge.

**Research question:**

To determine the learning curve for both interlaminar and transforaminal ELD in terms of operative time, and analyze factors that affect it.

**Materials and methods:**

Single-center retrospective study of 376 patients who underwent ELD between January 2013 and March 2024. In the cohort, 319 were in the interlaminar group and 57 in the transforaminal group. The learning curves were analyzed by CUSUM. The data regarding surgical technique, operative time, and postoperative outcome were analyzed.

**Results:**

The learning curve reached a plateau at 50 cases for the interlaminar technique and 23 cases for the transforaminal technique. Operative time was significantly lower for the transforaminal approach compared to the interlaminar approach: 69.18 ± 28.85 min versus 78.71 ± 28.86 min, p = 0.022. A second learning curve could not be demonstrated for the interlaminar approach in the long term. Operative time was influenced variably by factors such as age, gender, and level of herniated disc between the two techniques.

**Discussion and conclusion:**

Both the interlaminar and transforaminal ELD are minimally invasive techniques with different learning curves. The transforaminal approach shows a steeper learning curve and shorter operative time. The interlaminar approach did not show a second learning curve in the long term.

## Highlights

• Faster learning curve for the transforaminal technique 23 cases vs. interlaminar 50 cases.
• Significantly shorter operative time in the transforaminal group compared to interlaminar (69.18 vs. 78.71 min, p = 0.022).
•The interlaminar approach did not show a second learning curve in the long term.

## 1 Introduction

Lumbar disc herniation is considered one of the most common pathologies that we encounter in our clinical practice as spine surgeons (Schroeder et al., 2016). Until now, open microdiscectomy is considered the gold standard for treatment of this pathology (Dowling et al., 2024). However, in this technique we are still cutting the fascia, retracting the paraspinal muscles and removing part of the posterior bony elements before we enter the spinal canal and excise the disc herniations (Dowling et al., 2024). This procedure is therefore associated with some morbidities like wound complications, hematoma formation, infection, dura and nerve injury, instability and re-operation (Shriver et al., 2015; Bombieri et al., 2022). Endoscopic discectomy was later developed and refined to avoid some of these complications and is being gradually integrated by spine surgeons all over the world (Jang et al., 2021). However, endoscopic surgery might pose some challenges to new physicians, with a different approach to anatomy as well as complications (Lewandrowski et al., 2021; Yeung and Yeung, 2006). Therefore, it is of relevance to understand the learning curve of endoscopic techniques in lumbar discectomy as a guiding tool for upcoming practitioners. This learning curve can act as a benchmark for new surgeons to decrease complication rates and optimize patient safety.

## 2 Materials and Methods

### 2.1 Study design and participants

A retrospective study was conducted by reviewing the consecutive patients who had undergone endoscopic lumbar discectomy for disc herniation at Clemenceau Medical Center (CMC), a private tertiary referral hospital, between January 2013 and March 2024. All surgeries were performed by the same senior surgeon (MB), a senior spine surgeon with great experience in open and microdiscectomy. Inclusion criteria comprised individuals aged 18 years or above, who had undergone only endoscopic discectomy surgery for lumbar disc herniation. Exclusion criteria were prior lumbar surgery, and cases requiring endoscopic surgical treatment other than discectomy such as decompression or cyst resection.

### 2.2 Ethical consideration

This research is a retrospective study that was conducted respecting the ethical principles following the declaration of Helsinki developed originally in 1964. Confidentiality and anonymity of all data and participants were assured. Institutional Review Board (IRB) approval from the ethical committee of Clemenceau Medical Center was acquired (Ref: ERRC/RMRR/01/2024). This study was exempt from requiring informed consent from patients.

### 2.3 Data collection

Data was collected from patients’ medical records, imaging, operative reports, and endoscopic videos. The collected data includes: age, gender, BMI, surgery date, surgical level, technique used, operative time, herniated disc level, location, migration, and postoperative complications including nerve injury and dural tear.

The operation time was calculated from time of skin incision to time of closure.

### 2.4 Statistical analysis

The normality of the data was assessed using the Shapiro test. *t*-test and Mann-Whitney *U* test were used as appropriate to compare the mean of the dependent variable between two categories, and Kruskal-Wallis H Test was used to compare the means between more than two categories. Chi-square and Fisher's Exact test were used as appropriate for the bivariate analyses of the categorical variables. p < 0.05 was considered statistically significant.

All statistical analyses were performed using IBM SPSS Statistics for Windows, Version 27.0. Armonk, NY: IBM Corp.

Operative time was used as the main indicator of the learning curve. The learning curve of each technique was analyzed using the CUSUM methods, with the cases ordered chronologically. CUSUM refers to cumulative sum analysis, which is a sequential analysis technique of certain events typically used to monitor change detection (Novoa and Varela, 2020).

The CUSUM was based on the operative time defined by the formula **CUSUM = ∑ᵢ**_**=1**_^**n**^ **(Xᵢ − U)**, where Xi indicates the operation time of each case, U indicates the mean operation time of all cases, and n denotes the consecutive case number. This means that the sequential analysis of consecutive cases is monitored for a significant change in operative time with respect to the mean operative time. In CUSUM, when the significant change is detected, the curve shifts from positive to negative at this point (Novoa and Varela, 2020). In this way, this sequential technique is useful to detect leaning curve plateau after which the operative time is not significantly affected with sequential addition of cases.

### 2.5 Surgical procedure

All patients underwent discectomy, with no sequestrectomy procedures performed.

#### 2.5.1 Interlaminar technique

The patient is placed in the prone position under general anesthesia. Positioning of the patient and localization of the surgical level under fluoroscopy. The surgeon stands on the affected side. A one centimeter incision is made over the interlaminar window and using the Riwo spine system, a dilator is inserted up to the level of the ligamentum flavum followed by insertion a working channel. The endoscope is then inserted in the working channel. A detailed surgical approach is described in the literature (Won et al., 2021). After discectomy is performed, the working channel is removed and the skin is sutured.

#### 2.5.2 Transforaminal technique

The patient is placed in the prone position under general anesthesia. Positioning of the patient and localization of the surgical level was done under fluoroscopic control. The surgeon stands on the affected side. The entry point is based on the level of the discectomy and is approximately 10–14 cm lateral to the midline. Under fluoroscopic control, the needle is inserted through the foramen, followed by the dilator and the RIWO spine system endoscope is introduced. A detailed approach is described in the literature (Lee and Ahn, 2021). After discectomy is performed, the entire working channel is removed and the skin was sutured.

## 3 Results

### 3.1 Demographic characteristics

The total sample included 376 patients. It consisted of 212 males and 164 females, with an average age of 43.41 ± 14.18 years. The main technique used was interlaminar (84.8%), and the most common herniated disc level, location, and migration were L5-S1 (49.5%), paracentral (77.3%), and middle (52.2%) respectively. The mean operative time in minutes was 77.26 ± 29. The remaining demographic and operative characteristics data are shown in Table 1.

**Table 1:** Sample demographic and operative characteristics.

Characteristics	N (%) or Mean ± SD
*Age*	43.41 ± 14.18
*Gender*	
Males	212 (56.4%)
Females	164 (43.6%)
*BMI*	27.48 ± 4.21
*Operative time (min)*	77.26 ± 29
*Technique used*	
Interlaminar	319 (84.8%)
Transforaminal	57 (15.2%)
*Herniated disc level*	
L2-L3	7 (1.9%)
L3-L4	21 (5.6%)
L4-L5	162 (43.1%)
L5-S1	186 (49.5%)
*Herniated disc location (N=203)*	
Extraforaminal herniated disc	1 (0.5%)
Foraminal herniated disc	8 (3.9%)
Paracentral herniated disc	157 (77.3%)
Central herniated disc	37 (18.2%)
*Disc Migration (N=136)*	
High-Grade Up	3 (2.2%)
Low-Grade Up	7 (5.1%)
Middle	71 (52.2%)
Low-Grade Low	42 (30.9%)
High-Grade Low	13 (9.6%)

The interlaminar patient group was divided into 4 groups based on ascending chronological order (Table 2): first three groups 80 patients each and last group 79 patients. They were divided as the following: Group I included the first 80 cases [1, 80], Group II included cases [81, 160], Group III included cases [161, 240], and Group IV included cases [241, 319].

**Table 2:** Demographic and Operative Characteristics of the 4 interlaminar groups.

Characteristics	Groups				*p*-value
	I	II	III	IV	
*Age*	40.81 ± 12.37	44.39 ± 13.88	45.82 ± 15.06	43.87 ± 15.29	0.178
*Gender*					
Males	45 (24.5%)	44 (23.9%)	46 (25%)	49 (26.6%)	0.822
Females	35 (25.9%)	36 (26.7%)	34 (25.2%)	30 (22.2%)	
*BMI*	27.56 ± 3.90	28.16 ± 4.47	26.77 ± 4.23	27.23 ± 4.09	0.21
*Operative time (min)*	94.25 ± 33.85	71.61 ± 19.26	70.53 ± 25.10	78.44 ± 29.20	**<0.001**
*Herniated disc level*					
L2-L3	0 (0%)	0 (0%)	2 (66.7%)	1 (33.3%)	
L3-L4	0 (0%)	4 (25%)	7 (43.8%)	5 (31.3%)	**<0.001**
L4-L5	11 (9.6%)	21 (18.4%)	38 (33.3%)	44 (38.6%)	
L5-S1	69 (37.1%)	55 (29.6%)	33 (17.7%)	29 (15.6%)	
*Herniated disc location (N=168)*					
Foraminal	0 (0%)	2 (50%)	2 (50%)	0 (0%)	
Paracentral	65 (50%)	37 (28.5%)	10 (7.7%)	18 (13.8%)	**0.01**
Central	14 (41.2%)	5 (14.7%)	6 (17.6%)	9 (26.5%)	
*Disc Migration (N=136)*					
High-Grade Up	0 (0%)	2 (100%)	0 (0%)	0 (0%)	
Low-Grade Up	1 (16.7%)	2 (33.3%)	1 (16.7%)	2 (33.3%)	
Middle	26 (44.1%)	11 (18.6%)	11 (18.6%)	11 (18.6%)	0.285
Low-Grade Low	11 (28.2%)	13 (33.3%)	5 (12.8%)	10 (25.6%)	
High-Grade Low	2 (16.7%)	5 (41.7%)	1 (8.3%)	4 (33.3%)	
Dural Tear					
No	78 (24.7%)	80 (25.3%)	79 (25%)	79 (25%)	0.621
Yes	2 (66.7%)	0 (0%)	1 (33.3%)	0 (0%)	
Nerve Injury					
No	79 (24.8%)	80 (25.2%)	80 (25.2%)	79 (24.8%)	1
Yes	1 (100%)	0 (0%)	0 (0%)	0 (0%)	

There was no difference in age, gender or BMI between the 4 groups, as shown in Table 2. The difference in operative time between the groups individually is shown in Table 3. This difference was only significant between group 1 and the remaining groups (p < 0.001), indicating no changes in the mean operative time, on the long term, after the first 80 cases.

**Table 3:** Comparison of operative time between the 4 groups individually.

Groups	Test Statistic	Std. Error	Std. Test Statistic	*p*-value
1–2	63.494	14.581	4.355	**<0.001**
1–3	70.494	14.581	4.835	**<0.001**
1–4	49.685	14.627	3.397	**<0.001**
2–3	7.000	14.581	0.480	0.631
2–4	−13.809	14.627	−0.944	0.345
3–4	−20.809	14.627	−1.423	0.155

### 3.2 Procedure type

The sample was also divided and compared according to the procedure type of interlaminar versus transforaminal (Table 4). No significant difference was found between both groups with regards to age, BMI, and even complication rates. The mean operative time of the interlaminar group was 78.71 ± 28.86 min which is significantly higher than that of the transforaminal group at 69.18 ± 28.85 min (p = 0.022).

**Table 4:** Comparison of interlaminar versus transforaminal groups.

		Technique		*p*-value
		Interlaminar	Transforaminal	
Age		43.72 ± 14.25	41.63 ± 13.74	0.305
BMI		27.43 ± 4.195	27.70 ± 4.301	0.414
Operative Time (min)		78.71 ± 28.86	69.18 ± 28.85	**0.022**
Gender	Male	183 (57.5%)	29 (50%)	0.23
	Female	135 (42.5%)	29 (50%)	
Herniated Disc Level	L2-L3	3 (0.9%)	4 (7%)	
	L3-L4	16 (5%)	5 (8.8%)	**<0.001**
	L4-L5	114 (35.7%)	48 (84.2%)	
	L5-S1	186 (58.3%)	0 (0%)	
Herniated Disc Location^a^	Extraforaminal	0 (0%)	1 (2.9%)	
	Foraminal	4 (2.4%)	4 (11.4%)	**0.005**
	Paracentral	130 (77.4%)	27 (77.1%)	
	Central	34 (20.2%)	3 (8.6%)	
Disc Migration^b^	High-Grade Up	2 (1.7%)	1 (5.6%)	
	Low-Grade Up	6 (5.1%)	1 (5.6%)	
	Middle	59 (50%)	12 (66.7%)	0.463
	Low-Grade Low	39 (33.1%)	3 (16.7%)	
	High-Grade Low	12 (10.2%)	1 (5.6%)	
Dural Tear	No	316 (99.1%)	56 (98.2%)	0.581
	Yes	3 (0.9%)	1 (1.8%)	
Nerve Injury	No	318 (99.7%)	57 (100%)	0.672
	Yes	1 (0.3%)	0 (0%)	

a *N* = 203.

b *N* = 136.

Due to lack of data, herniated disc location was only documented in 203 of the cases, and disc migration in only 136 of the cases. The transforaminal procedure shows significant lower operative time (69.18 ± 28.85 min) in comparison to the interlaminar procedure (78.71 ± 28.86 min) (p = 0.022). A significant difference was seen in the interlaminar group, herniated disc level at L5-S1 was 58.3%, in comparison to the transforaminal group where herniated disc level at L4-L5 was 84.2% (p < 0.001). Central discs were located significantly more in interlaminar group with 20.2% than in transforaminal group with 8.6%. Extraforaminal and foraminal discs were located more in transforaminal group with 2.9% and 11.4% respectively, than in interlaminar group with 0% and 2.4% respectively. Paracentral discs were located at an approximately similar rate in both groups with 77.4% in interlaminar group and 77.1% in transforaminal group (p = 0.005).

### 3.3 Operative time in each procedure type

**Fig. 1:**
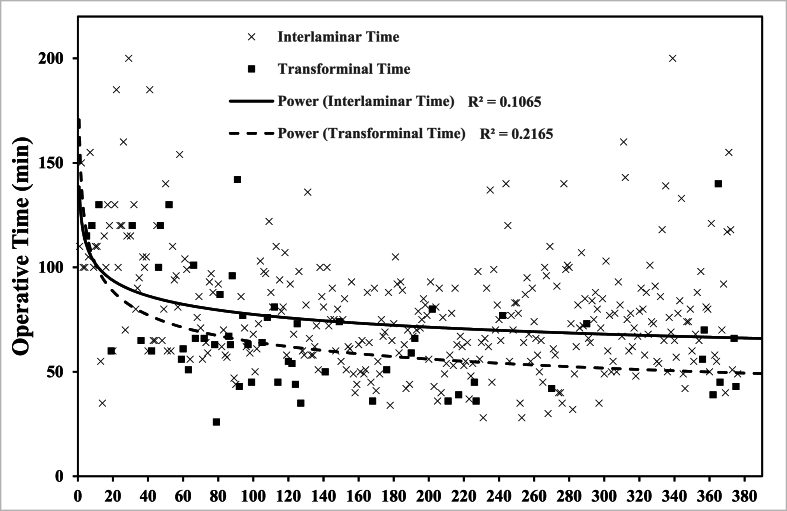
Trendline of Op Time of all the sample (interlaminar vs transforaminal).

Fig. 1 plots the operative time (min) against the case numbers in chronological order of all the sample. It shows the best-fit curve of both interlaminar and transforaminal operations. The transforaminal group shows lower operative time in comparison to the interlaminar group throughout the sample size.

**Fig. 2:**
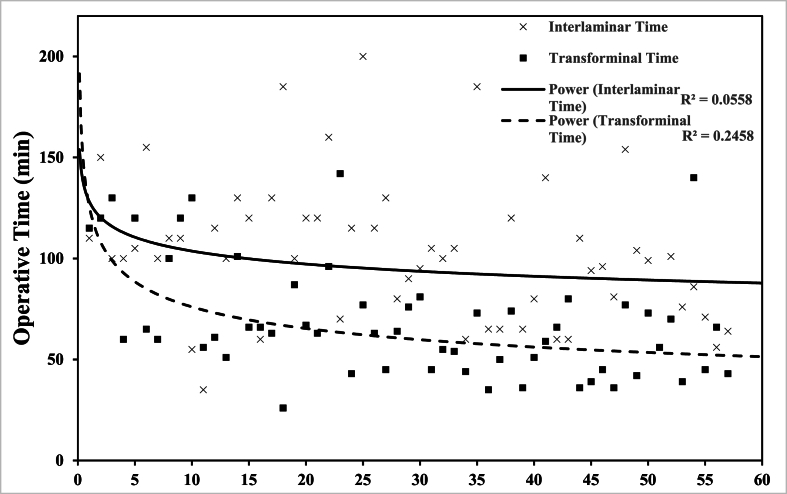
Trendline of Op Time of the first 57 cases of each technique (interlaminar vs transforaminal.

Taking into consideration the lower sample size of the transforaminal group, Fig. 2 shows the trendline of the operative time between both procedures using chronologically the first 57 cases of each separate technique. The operative time was still shown to be lower in the transforaminal group time in comparison to the interlaminar group.

### 3.4 Operative time

Table 5 shows the effect of different population characteristics on the operative time in the interlaminar group. BMI, gender, and operative complications had no significant effect on the operative time (p > 0.05). Patients under 65 years had significantly (p = 0.046) lower operative time (77.52 ± 28.28 min) than patients over 65 years of age (89.03 ± 32.12 min).

**Table 5:** Effect of population characteristics on operative time in the interlaminar group.

Interlaminar (N = 319)			
		OR Time (min)	*p*-value
Age	Age<65 (N = 286)	77.52 ± 28.28	**0.046**
	Age ≥ 65 (N = 33)	89.03 ± 32.12	
BMI	Non-Obese (N = 241)	77.84 ± 28.63	0.214
	Obese (N = 78)	81.38 ± 29.58	
Gender	Male (N = 184)	80.91 ± 30.54	0.15
	Female (N = 135)	75.71 ± 26.20	
Herniated Disc Level	L2-L3 (N = 3)	159 ± 35.53	**<0.001**
	L3-L4 (N = 16)	75 ± 26.95	
	L4-L5 (N = 114)	84.16 ± 28.23	
	L5-S1 (N = 186)	74.39 ± 27.00	
Herniated Disc Location^a^	Foraminal (N = 4)	56.5 ± 30.91	0.124
	Paracentral (N = 130)	84.67 ± 30.21	
	Central (N = 34)	87.5 ± 30.86	
Disc Migration^b^	High-Grade Up (N = 2)	90 ± 21.21	0.367
	Low-Grade Up (N = 6)	88.17 ± 7.36	
	Middle (N = 59)	82.17 ± 34.33	
	Low-Grade Low (N = 39)	84.1 ± 27.33	
	High-Grade Low (N = 12)	77.42 ± 36.14	
Dural Tear	No (N = 316)	78.73 ± 28.63	0.595
	Yes (N = 3)	76 ± 57.68	
Nerve Injury	No (N = 318)	78.37 ± 28.28	0.089
	Yes (N = 1)	185	

a 168.

b 118.

Interlaminar procedure performed at levels L3-L4, L4-L5 and L5-S1 showed different operative times of 75 ± 26.95 min, 84.16 ± 28.23 min, and 74.39 ± 27.00 min respectively. These values were significantly lower than interlaminar procedure performed at the level of L2-L3 which had a mean operative time of 159 ± 35.53 min (p < 0.001).

Data regarding herniated disc location was only available in 168 of the 319 patients (a = 168). No statistically significant difference between operative times with regards to herniated disc location (p = 0.124).

Data regarding disc migration was also only available in 118 of the 319 patients (b = 118). However, there was no statistically significant difference between operative times with regards to disc migration (p = 0.367).

Table 6 shows the effect of different population characteristics on the operative time in the transforaminal group. Age, BMI, herniated disc level, disc location, disc migration, and operative complications had no significant effect on the operative time (p > 0.05). Male patients had a significantly longer mean operative time of 77.82 ± 32.87 min compared to female patients with a mean operative time of 60.83 ± 21.82 min (p = 0.045).

**Table 6:** Effect of population characteristics on operative time in the transforaminal group.

Transforaminal (N = 57)			
		OR Time (min)	*p*-value
Age	Age<65 (N = 54)	68.87 ± 28.12	0.853
	Age ≥ 65 (N = 3)	74.67 ± 48.08	
BMI	Non-Obese (N = 38)	65.24 ± 22.64	0.472
	Obese (N = 19)	77.05 ± 37.93	
Gender	Male (N = 28)	77.82 ± 32.87	**0.045**
	Female (N = 29)	60.83 ± 21.82	
Herniated Disc Level	L2-L3 (N = 4)	92.25 ± 38.95	0.327
	L3-L4 (N = 5)	73 ± 36.02	
	L4-L5 (N = 48)	66.85 ± 27.06	
Herniated Disc Location^a^	Extraforaminal (N = 1)	115	0.149
	Foraminal (N = 4)	100 ± 36.05	
	Paracentral (N = 27)	74.93 ± 30.71	
	Central (N = 3)	48.67 ± 21.93	
Disc Migration^b^	High-Grade Up (N = 1)	130	0.104
	Low-Grade Up (N = 1)	43	
	Middle (N = 12)	64.83 ± 27.83	
	Low-Grade Low (N = 3)	98.33 ± 23.11	
	High-Grade Low (N = 1)	43	
Dural Tear	No (N = 56)	69.09 ± 29.11	0.632
	Yes (N = 1)	74	
Nerve Injury	No (N = 57)	69.18 ± 28.85	–

a 35.

b 18.

### 3.5 CUSUM analysis of the learning curve

**Fig. 3:**
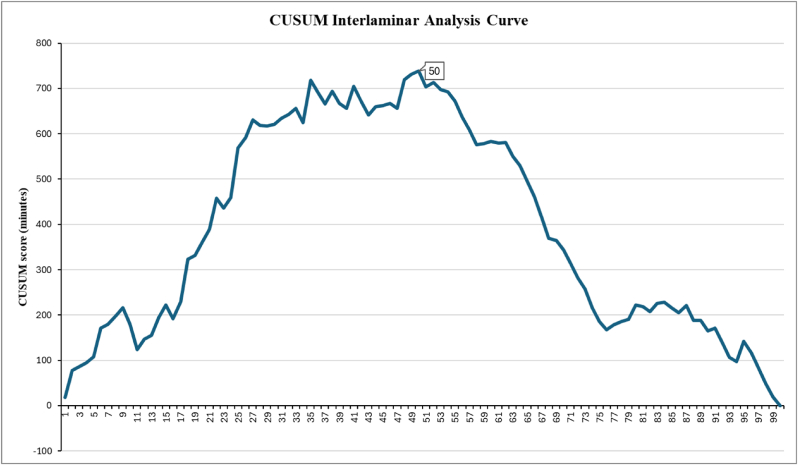
CUSUM Analysis Curve of the interlaminar technique. The cut-off point was 50 cases, after which the slope of the curve turned from positive to negative.

**Fig. 4:**
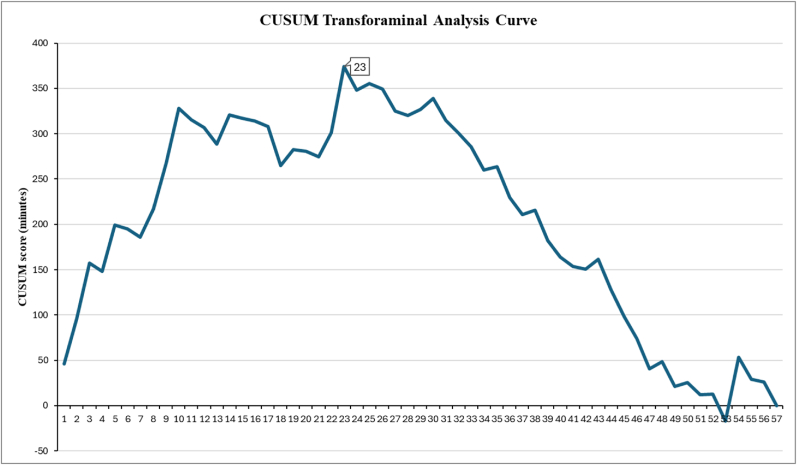
CUSUM Analysis Curve of the transforaminal technique. The cut-off point was 23 cases, after which the slope of the curve turned from positive to negative.

CUSUM analysis of the learning curve of the two techniques was used to determine the 2 scatterplots shown in Fig. 3, Fig. 4. For the interlaminar technique, as previously shown, the learning curve plateaus in the first 80 cases, and with no secondary learning curve, the primary improvement period would remain consistent regardless of how many cases are included above the 80 cases, so 100 cases were included in the CUSUM analysis (Fig. 3). The scatterplot revealed a cut-off point at 50 cases, after which the slope of the curve switched from positive to negative. This means that the learning curve plateaus at 50 cases while using the interlaminar technique. As for the transforaminal technique, the scatterplot (Fig. 4) reveals that the cut-off point was at 23 cases, meaning that the learning curve plateaus at 23 cases while using the transforaminal technique.

## 4 Discussion

The goal of discectomy is to decompress neural tissue while preserving spine stability. Minimally invasive surgery, including endoscopic spine surgery, was introduced in the past three decades to improve functional outcomes by reducing damage to paraspinal tissues and spinal ligaments (Jang et al., 2021). As a result, many spine surgeons are adopting endoscopic lumbar discectomy as an alternative to open procedures (Lewandrowski et al., 2021). However, as stated earlier, endoscopic surgery possesses a different approach to anatomy and technique, and might pose a challenge to new physicians (Yeung and Yeung, 2006). Henceforth, the learning curve of endoscopic lumbar discectomy using the interlaminar and transforaminal technique each was described in this study.

Previous retrospective studies have been described in the literature tackling the learning curve of endoscopic lumbar discectomy (Lee and Lee, 2008; Wu et al., 2016; Hsu et al., 2013a; Wang et al., 2013); however, these studies had under 100 patients or spanned a selection of patients over a few years. To our knowledge, this is the largest study done for the learning curve of endoscopic lumbar discectomy in the Middle East, comprising of 376 patients over a period of 10 years.

In the studies mentioned, operative time was the main indicator for learning curve progression. The mean operative time was therefore chosen as the main indicator of learning curve progression in our study.

In the interlaminar group, categorizing the population into 4 groups based on chronological cases revealed that the first 80 cases had significantly more operative time than the remaining groups without differences between the remaining groups. This indicates that the primary learning curve is attained in the first 80 cases and there is no secondary learning curve. Therefore, CUSUM analysis of the first 100 cases was performed to reveal the learning curve peak point.

CUSUM refers to cumulative sum analysis and was introduced by Page in 1954, with several modifications added afterwards (Novoa and Varela, 2020). This method of analysis is used to detect changes in sequential events. The graph reveals the result of cases in chronological order, with positive and negative outcome for “out of control” versus “in control” cases respectively, based on defined benchmark controls. The goal is the target value at which point the graph leans from positive to negative (Novoa and Varela, 2020).

The CUSUM result in our study showed that the learning curve peak point using the interlaminar technique was attained at the 50th case, after which the slope of the curve went from positive to negative. Anh et al. (Ahn et al., 2021) conducted a systematic review of the learning curve of interlaminar endoscopic lumbar discectomy and included a total of 6 studies done in China, Switzerland, Russia, Korea and Italy. The mean operative time was also the most commonly used measure for the learning curve. The cut-off value for the learning curve in these studies ranged from 10 to 43 cases; however, not all the studies used a cumulative sum analysis. In our study, the cut-off point was revealed to be slightly higher at 50 cases. It is important to know that the surgeon and the operating room staff members, as well as the operating room set up and materials available, may all play a part in operative time and hence the learning curve.

The CUSUM analysis on transforaminal cases in our study showed that the learning curve peak point was attained at the 23rd case, after which the slope went from positive to negative. Another systematic review by Anh et al. (Ahn et al., 2020) tackled the learning curve for transforaminal endoscopic lumbar discectomy and included a total of 10 articles done in China, Spain, Korea, Israel, Thailand and Taiwan. The main indicator used to measure the learning curve was the operative time, and the cut-off value of the learning curve ranged from 10 to 72 cases. However, most of the studies had a range of 10–20 cases, and the study with the cut-off point at 72 cases did not only measure operative time, but also success rate of 90%, which was not the target of our study.

A difference in operative time was observed in our study between the interlaminar and transforaminal procedure. The interlaminar technique was associated with a significant increase in operative time compared to the transforaminal technique. Taking into consideration the difference in sample size between both groups, comparing the two techniques in only the first 57 cases revealed that the interlaminar technique had longer operative time as well. In concordance with our results, a study done by Hsu et al. (2013b) on the learning curve of full endoscopic discectomy revealed that the transforaminal procedure required less operative time than the interlaminar technique. In contrast, a metanalysis done by Jitpakdee et al. (2023) where interlaminar and transforaminal endoscopic lumbar discectomy were compared revealed that the transforaminal group had a significant increase in operative time in comparison to the interlaminar group. However, a subanalysis of the groups did show that the results differ according to the level of the discectomy. This might explain the contradiction in the results, where the transforaminal technique shows less operative time in the upper lumbar spine while the interlaminar technique exhibits lower operative time in the lower lumbar spine.

Our study revealed that obesity had no significant effect on operative time in both the interlaminar and transforaminal group. In a metanalysis done by Feng et al. (2024), the outcomes of obese patients were compared to non-obese patients when endoscopic lumbar discectomy was performed. In the mentioned study, obese patients had significantly longer operative time than non-obese patients, and this was attributed to possible technical difficulties and variations in anatomy. Further studies might prove useful in elucidating the role of obesity on the perioperative duration in endoscopic lumbar discectomy.

An increase in age has been shown to affect lumbar spine morphology (Benoist, 2003). Older age is associated with degeneration and stenosis of the neural foramina, and the loss of disc height leads to a decrease in the interlaminar window (Benoist, 2003; Yan et al., 2018). These changes can theoretically increase the technical difficulty of the operation, and henceforth increase the surgical time. In a study done by Son et al. (2023), a comparison of the effect of age on the results of transforaminal endoscopic lumbar discectomies was correlated with no significant difference with regards to operative time. The results of our study as well reveal that age ≥ 65 years had no significant effect on the operative time of the transforaminal group. However, patients older than 65 years did exhibit longer operative times when the interlaminar technique was used. To our knowledge, there is no study in the literature that addresses the age-related morphology effect on the operative time in interlaminar lumbar discectomy. Using the interlaminar technique, cranial laminotomy and ligamentum flavum resection is often performed to enlarge the interlaminar window (Chen et al., 2020). Therefore, one might speculate an increase in operative time when laminotomy is necessary in performing the surgery.

According to gender, there was no significant difference in operative times between males and females in the interlaminar group. On the other hand, male patients had a significantly longer mean operative time of 77.82 ± 32.87 min compared to female patients with a mean operative time of 60.83 ± 21.82 in the transforaminal group. There is a paucity of evidence in the literature to surmise the effect of gender on the operative time in endoscopic spine surgery. A study done by Parrish et al. (2020) compared gender effect on minimally invasive transforaminal lumbar interbody fusion. This study showed no difference in operative time between males and females. A study done on endoscopic lumbar discectomy is needed to further evaluate the effect of gender in this case.

Anatomically, the upper lumbar spine differs from the lower lumbar spine, with smaller vertebra and intervertebral discs in the upper portion, and a shallow lateral recess (Wang et al., 2022). The narrow interlaminar window in the upper spine therefore might be a hindrance in performing interlaminar endoscopy. However, the foramina in the upper lumbar spine are wider and larger, theoretically ameliorating the passage of endoscopic instruments, thus making transforaminal endoscopy technically less difficult (Wang et al., 2022). In our study, in the transforaminal group, there was no difference in operative time with regards to the level of surgical discectomy. In contrast, operative time at the L2-L3 level using the interlaminar technique was significantly longer than lower lumbar levels, which may be caused by the narrow interlaminar window. In their study, Yuan et al. (2024) compared the characteristics of upper and lower lumbar levels during endoscopic discectomy, where the upper lumbar levels did indeed have longer operation time, however this difference was not significant. Based on these findings, it is may be recommended for the new surgeons learning endoscopic lumbar discectomy to start on interlaminar discectomy in the lower lumbar levels.

Herniated discs can be classified as central, paracentral, foraminal and extraforaminal based on anatomical landmarks (Lee and Lee, 2016). Central discs were more common in the interlaminar group than in the transforaminal group (20.2% vs 8.6%), whereas foraminal and extraforaminal discs were more common in the transforaminal group than interlaminar group (11.4% and 2.9% vs 2.4% and 0% respectively). Paracentral herniated discs were the most common encountered pathology with similar rates in both interlaminar and transforaminal group (77.4% and 77.1% respectively). However, using both the transforaminal and interlaminar technique in our study did not reveal any significant differences in operative time according to the herniated disc location. There is a paucity of studies in the literature that tackle the possible effect of herniated disc location on the technique used. There were no cases of extraforaminal discs using the interlaminar group in our study. Theoretically, extraforaminal discs pose a challenge using the interlaminar technique without removal of the facet joint (Yadav et al., 2016). Therefore, the removal or preservation of facet joints and the difficulties that the surgeon can face in this case can theoretically increase the operative time. Further studies are required to significantly correlate operative duration according to the location of herniated discs in endoscopic discectomy.

In our study, operative time did not show any correlation with disc migration in the transforaminal nor in the interlaminar group. More studies may be needed to confirm this finding.

Finally, It is important to recognize that other techniques such as endoscopic decompression cyst resection are more complex than simpler discectomy cases and may represent different learning curve.

## 5 Conclusion

Due to its minimally invasive nature, endoscopic lumbar discectomy is becoming a popular technique for the treatment of herniated discs of the lumbar spine. The operation can be performed using either the interlaminar or transforaminal technique. This study assessed the learning curves for interlaminar and transforaminal techniques, revealing cut-off points of 50 and 23 cases, respectively, highlighting the faster learning curve of the transforaminal method. Additionally, the transforaminal approach demonstrated shorter operative times compared to the interlaminar technique. Factors such as age, gender, herniation level, and BMI showed variable impacts on operative time, underscoring the need for individualized consideration in surgical planning.

### 5.1 Limitation

The study relies solely on operative time as an indicator for learning curve progression. There are other metrics, such as complication rates (other than nerve injury and dural tear), patient outcomes, or surgeon-reported confidence, were not included. These different metrics might reveal different learning curves. Furthermore, the study reflects the learning curve of a single surgeon. In addition, the study is based on data from a single medical center, and variations in equipment, staff experience, or patient demographics in other settings could yield different learning curve thresholds. In this respect, a single surgeon and single center retrospective study might not be generalizable to all surgeons. The relatively low number of transforaminal cases over the study period should be considered when interpreting the findings.

## Funding

No funding was received for this research.

## Declaration of competing interest

The authors declare that they have no known competing financial interests or personal relationships that could have appeared to influence the work reported in this paper.
